# First record of the genus *Arria* (Mantodea, Haaniidae, Arriini) from Thailand, with the description of a new species of moss-dwelling praying mantis

**DOI:** 10.3897/zookeys.1028.62347

**Published:** 2021-04-05

**Authors:** Thornthan Unnahachote, Evgeny Shcherbakov, Nantasak Pinkaew

**Affiliations:** 1 Department of Entomology, Faculty of Agriculture at Kamphaeng Saen, Kasetsart University, Nakhon Pathom, 73140 Thailand Kasetsart University Nakhon Prathom Thailand; 2 Department of Entomology, Faculty of Biology, Lomonosov Moscow State University, Leninskie Gory st. 1 bldg 12, Moscow 119991, Russia Lomonosov Moscow State University Moscow Russia

**Keywords:** Camouflage, predatory insect, Southeast Asia, taxonomy

## Abstract

*Arria muscoamicta* Unnahachote & Shcherbakov, **sp. nov.** is described based on a male from central Thailand. This is the first record of *Arria* Stål, 1877 from the country. The new species is closely allied to *A. leigongshanensis* (Ge & Shen, 2008) from China, differing by the absence of prozonal tubercles, the elongated pronotum, nine tibial anteroventral spines, and the truncated hindwings. The new species is a moss-camouflaging mantis living at high altitude. The taxonomic problems of the genus are briefly discussed.

## Introduction

There are many genera of praying mantises from both the Old and the New World, and some members are camouflaged as moss, such as the following genera: *Astape* Stål, 1877, *Haania* Saussure, 1871, *Majangella* Giglio-Tos, 1915, and *Pseudopogonogaster* Beier, 1942. These, as well as others, are colloquially referred to as “moss mantises”. Almost all of them have evolved special morphology, such as spines, lobes, and tubercles on their bodies, which aid in their camouflage on moss beds (Beier 1952; Rivera et al. 2011; Svenson and Vollmer 2014). Among the least studied of the genera that include moss-camouflaging species is the genus *Arria*, which was described by Stål (1877) with *Arria cinctipes* Stål, 1877 as its type species (type locality “India orientalis”). Species of *Arria* exhibit a strong sexual dimorphism: the male has well-developed wings reaching beyond the tip of the abdomen, while the female is completely apterous. In addition, they live at high elevations, and the ootheca has a small number of eggs, making it difficult to obtain specimens from field surveys (Ge and Chen 2008; Zhu et al. 2012). After the most recent taxonomic changes (Schwarz and Roy 2018; Wang et al. 2021), there are currently eight species belonging to the genus: *Arria cinctipes* and *Arria meghalayensis* (Mukherjee, 1995) from India: “India orientalis” and Meghalaya, respectively; and *Arria oreophilus* (Tinkham, 1937), *Arria pallida* (Zhang, 1987), *Arria brevifrons* (Wang & Bi, 1991), *Arria sticta* (Zhou & Shen, 1992), *Arria leigongshanensis* (Ge & Chen, 2008), and *Arria pura* Wang & Chen, 2021 from China: Sichuan, Yunnan, Zhejiang, Hainan, and Guizhou, respectively. However, only one species, *A. leigongshanensis*, was known as being a moss-camouflaging species. Here we describe a new species closely related to *A. leigongshanensis* from high-elevation, mossy forests in central Thailand, Nakhon Nayok province, representing the first report of the genus from the country.

## Materials and methods

The male holotype was collected at a light trap and preserved in a freezer before being pinned on a mounting block and dried. Five nymphs were found on separate occasions by visual inspection in the moss close to where the holotype was collected. The holotype is deposited at the Thailand Natural History Museum (**THNHM**). The nymphs could not be preserved.

For genitalia preparations, the tip of abdomen was separated from the specimen and macerated in 10% potassium hydroxide (KOH) solution, then rinsed with demineralised water and placed in glycerine for dissection. Afterwards it was placed in a genital vial with glycerine for long-term preservation and pinned together with the holotype.

Observation of the external structures and male genitalia were made with an Optika microscope (Optika Microscopes, Italy). Live photographs of the adult were taken by W. Pathomwattananuruk with a Nikon AF-S Micro Nikkor 60 mm lens attached to a Nikon D7000 camera. Live photograph of the nymph was taken by W. Khaikaew with an AF-S Micro 60 mm f/2.8G lens attached to a Nikon D610. Male genitalia photographs were taken with a Leica S8 APO stereomicroscope equipped with a Leica MC170 HD camera module. The classification system is according to Schwarz and Roy (2019). The morphological nomenclature and standards of measurement follow Brannoch et al. (2017), Schwarz and Roy (2019), and Vermeersch (2018).

### Abbreviations

**AL** Ala length

**AvS** Anteroventral spine

**CfW** Costal field width of tegmen

**DS** Discoidal spine

**F** Femur

**HW** Head width

**MsFL** Mesofemur length

**MstL** Mesotarsus length

**MsTL** Mesotibia length

**MtFL** Metafemur length

**MttL** Metatarsus length

**MtTL** Metatibia length

**MzL** Metazone length

**PCL** Procoxa length

**PFL** Profemur length

**PL** Pronotum length

**PnW** Pronotum narrow width

**PtL** Protarsus length

**PTL** Protibia length

**PvS** Posteroventral spine

**PW** Pronotum width

**PzL** Prozone length

**T** Tibia

**TgL** Tegmen length

**TL** Total length

### Depositories

**GUGC** Institute of Entomology Guizhou University, Guiyang, China;

**SMNK**Staatliches Museum fur Naturkunde, Karlsruhe, Germany;

**THNHM** Thailand Natural History Museum, Pathum Thani, Thailand.

## Systematic accounts

### Order Mantodea Burmeister, 1838

**Family Haaniidae Giglio-Tos, 1915**

**Subfamily Haaniinae Giglio-Tos, 1915**

**Tribe Arriini Giglio-Tos, 1919**

**Genus *Arria* Stål, 1877**

#### Arria muscoamicta

Taxon classificationAnimaliaMantodeaMantidae

Unnahachote & Shcherbakov sp. nov.

325A00F4-E818-57EF-8463-1867DFF587C1

http://zoobank.org/212326E3-D5A6-445A-AC4F-9A2722B9B48C

**Figure 1.**
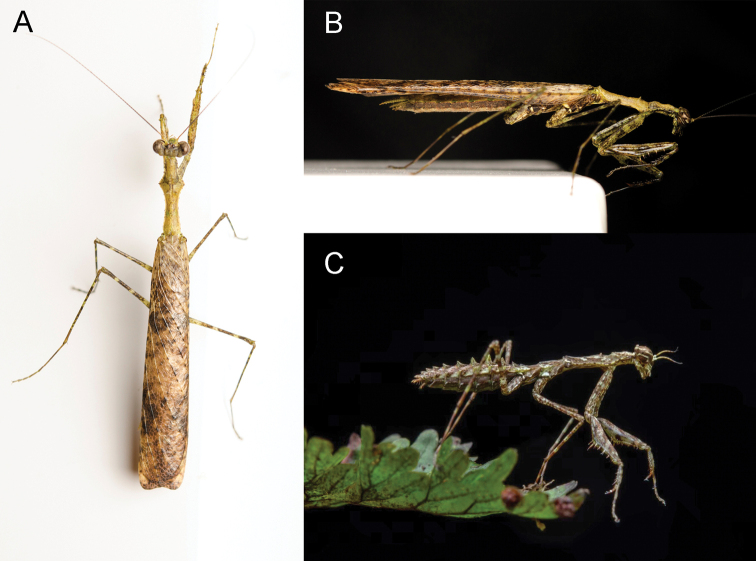
*Arria muscoamicta* sp. nov. in life aspect **A** adult male (the holotype), dorsal view **B** adult male (the holotype), lateral view **C** male nymph. **A, B** W. Pathomwattananuruk, published with permission **C** W. Khaikaew, published with permission.

**Figure 2.**
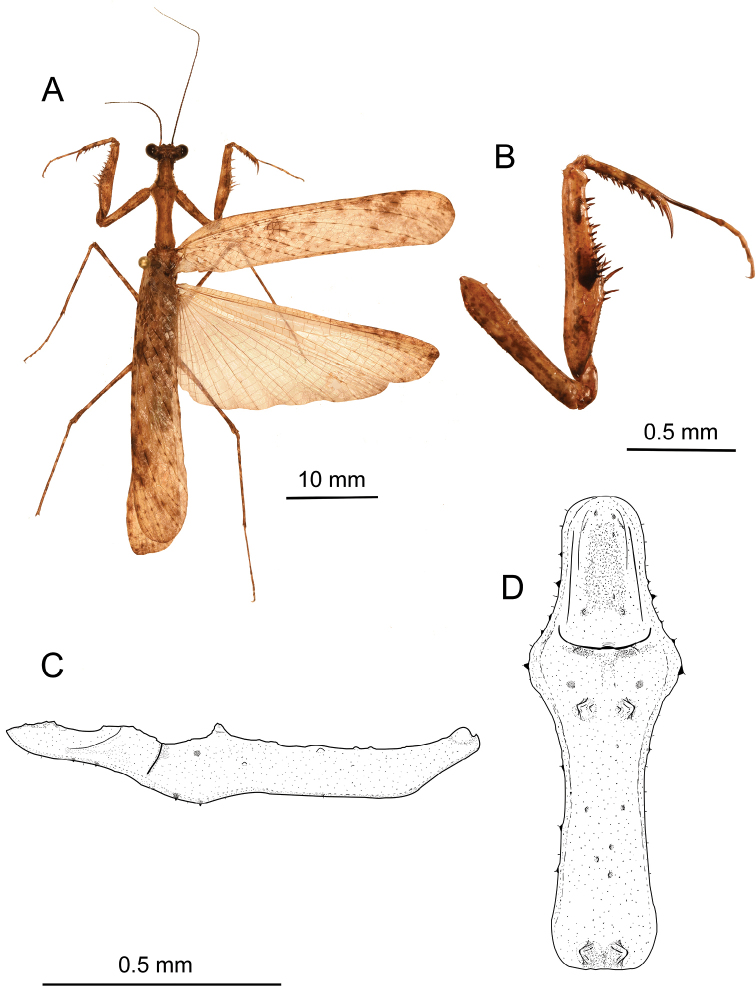
*Arria muscoamicta* sp. nov. **A** dorsal habitus **B** anterior side of prothoracic leg **C, D** pronotum in lateral and dorsal views, respectively.

**Figure 3.**
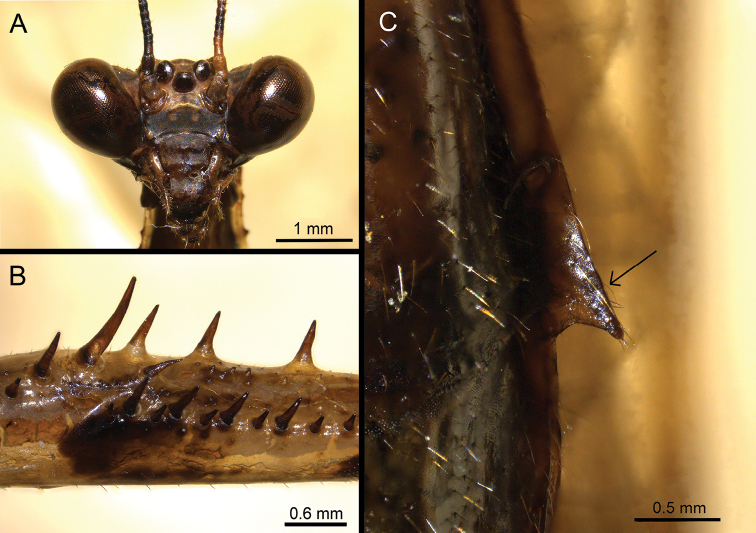
*Arria muscoamicta* sp. nov. **A** head in frontal view **B** ventral view of prothoracic femur **C** lateral lobe of abdominal tergite.

**Figure 4.**
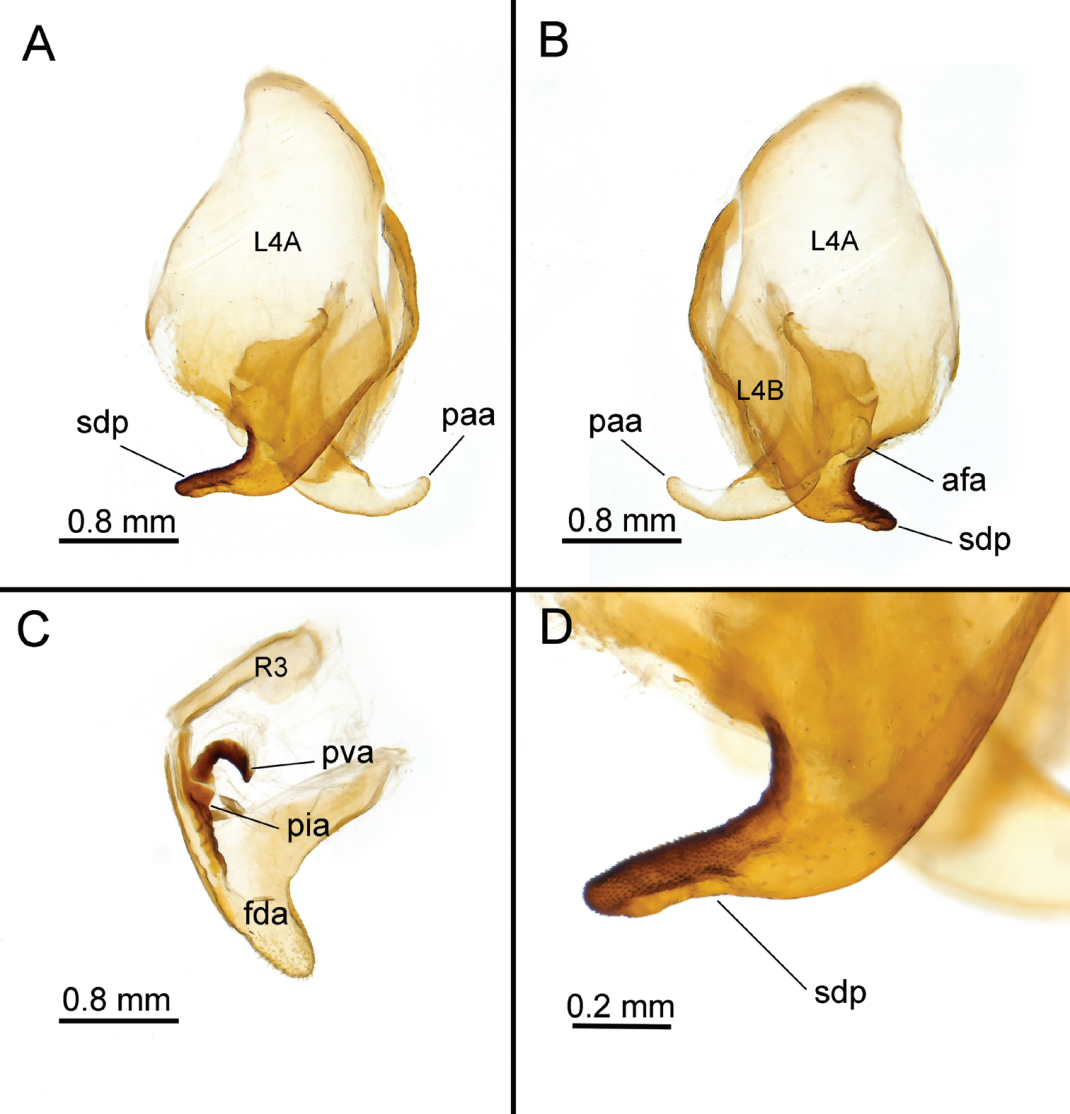
*Arria muscoamicta* sp. nov., male genitalia **A, B** left complex in ventral and dorsal perspectives, respectively **C** right phallomere in ventral perspective **D** close up of sdp.

Figures 1 , 2 , 3 , 4

##### Type material.

***Holotype*.** Thailand – Nakhon Nayok Province • 1 ♂; Mueang district, Hin Tung subdistrict; 14°21'56"N, 101°24'1"E; 01.IX.2018; alt. 1,240 m; W. Pathomwattananuruk leg.; THNHM-I-23353.

##### Comparative material.

*Arria* sp. Laos – Bokeo • 1 ♂; Van Pak Len, an Brücke Goldenes Dreieck; 20°12'36"N, 100°3'36"E; 01.IX.2018, IV.1979; H. Lehmannsen leg. (SMNK).

##### Comparative photographic material.

*Arria leigongshanensis* (Ge & Chen, 2008). Holotype; CHINA – Guizhou • 1 ♂; Leishan, Leigongshan; 13.IX.2005; Song Qiong-Zhang leg. (GUGC).

##### Differential diagnosis.

*A. muscoamicta* sp. nov. is similar to the type species of *Arria*, *A. cinctipes*, in foreleg armament and shape of the prothorax and wings; it fits the current concept of the genus *Arria* (but see Discussion).

*Arria muscoamicta* sp. nov. can be distinguished from the most similar species, *A. leigongshanensis*, by the following characters: 1) pronotum distinctly longer; MzL/PzL = 1.97 [vs MzL/PzL = 1.24], 2) prozone without distinct pair of conical spines posteriorly [vs with distinct pair of conical spines posteriorly, anteriad of supracoxal sulcus], 3) foretibia have nine anteroventral spines [vs 11–13 anteroventral spines], 4) apical lobe of hindwing almost truncated [vs more or less parabolic].

*Arria muscoamicta* sp. nov. can also be easily distinguished from *A. cinctipes* by the following characters: 1) six tibial posteroventral spines [vs seven tibial posteroventral spines], 2) lack of a pair of small conical tubercles in prozone posteriorly [presence of a pair of small conical tubercles in prozone posteriorly]; from *A. meghalayensis* by six tibial posteroventral spines [vs seven tibial posteroventral spines]; from *A. oreophilus* by following characters: 1) present of conical tubercles on dorsal surface of pronotum [vs lack of conical tubercles, relatively smooth in male], 2) forewing not narrows distally [vs forewing narrows distally]; from *A. sticta* and *A. pallida* by the apex of hindwing more or less truncate [vs pointed apex].

##### Etymology.

The name of the species means “clothed by moss” in Latin and refers to the moss-like colouration and morphology of the adults and especially the nymphs.

##### Description.

**Adult male. *Head*** (Fig. 3A). Wider than long, compound eyes strongly protruded antero-laterally, rounded. Ocellar tubercle elevated. Ocelli large. Lateral lobes of vertex elevated higher than median lobe. Antenna filiform with fine setae, longer than pronotum length, almost entirely dark except pedicel and proximal segments, which are pale green (discolouration in dried specimen). Lower frons (frontal shield) transverse, surface smooth, anterior margin and posterior margin relatively arched. Postfrontal sulcus noticeably elevated. Juxtaocular bulges distinctly protruded, higher than vertical dorsal line. Clypeus with short medial ridge and labrum entirely smooth.

***Pronotum*** (Fig. 2C, D). More or less slender, longer than forecoxa length, ratio of MzL/PzL = 1.97. Supracoxal dilation very prominent. Lateral margin with denticles strongly present at supracoxal dilation and the prozone, less prominent at metazone, with setae along the margin. Dorsal line of prozone concave in the middle. Dorsal surface more or less tuberculate, with two pairs of strong conical tubercles on metazone, anterior pair a little bit larger than posterior pair, and with a small tubercle laterad of each conical tubercle, while only small tubercles present in the prozone. Pair of small depressions present at anterior half of metazone posteriorly of supracoxal sulcus. Cervicalia complete. Anterior and posterior ventral cervical sclerites similar in size and shape, non-interrupted. Intercervical sclerites connected to those on opposite side, transverse, margin elevated, distinctly concave at the middle, anterior margin more or less angulated. Lateral cervical sclerites large, longer than wide, strongly concave along the side which close to ventral cervical sclerites.

***Prothoracic leg*** (Figs 2B, 3B). Coxa shorter than femur, internal surface somewhat smooth. Dorsal margin with a few short spines, with larger spines present in the proximal half, while very small or nearly absent at the middle and in the distal half. Ventral margin with small irregular denticles. Coxal lobes divergent, dorsal lobe a bit longer than ventral one. Femur with dorsal margin slightly S-shaped. Femoral brush ellipse-shaped. Tibial spine groove present near the middle of femur’s length. Anterior genicular lobe with a spine; posterior genicular lobe with a spine (absent on right side). Anterior side with distinctly two infuscate patches presents at middle of femur length and in the femoral brush area, respectively. Eleven or 12 AvS arranged as **iIiIiIiIiiiI** (**iIiIiIiIiii** on the right side), all AvS infuscate. Four DS, 3^rd^ largest, 1^st^ and 4^th^ somewhat equal in length. Four PvS equal in length. Ventral side with small blunt tubercles in the posterior half before 1^st^DS, row of smaller tubercles starting from 2^nd^DS to the distal half along anteroventral side, and a group of small acute tubercles present at medioventral to posteroventral area between 2^nd^ and 3^rd^PvS (Fig. 3B). Tibia with nine AvS elongating distally, 1^st^ smallest and 9^th^ longest respectively. Six PvS arranged as i_IIIII with gap between 1^st^PvS and base of tibia largest. First tarsomere of protarsus longer than remaining segments combined. Spinal formula: F = 4DS/11–12AvS/4PvS; T = 9AvS/6PvS.

***Meso- and metathoracic legs.*** Long and slender with fine setae, without dilations or projections. Femora with rounded genicular lobes each bearing a single short apical spine. Tibiae with two apical spines. First tarsomere of mesotarsus slightly longer than remaining segments combined. First tarsomere of metatarsus much longer than remaining segments combined.

***Flight organs.*** Forewing long, narrow, with rounded apex and covered by small setae. Costal area relatively narrow. Hindwing with almost truncated apex bearing small lobe anteriorly, protruding a little beyond forewing in resting position.

***Abdomen.*** Narrow, with small but distinct, acute lateral lobes on each abdominal tergite (Fig. 3C). Cerci cylindrical with numerous setae, last cercomere conical. Tergite X (supra-anal plate) transverse, covered by setae, posterior margin more or less rounded with small projection at the middle. Coxosternite IX (subgenital plate) longer than wide, two posterolateral ridges present on ventral side and forming base of styli ventrally. Posterior margin truncated. Ventral side with fine setae, much denser in posterior half and on styli.

***Genitalia*** (Fig. 4). Ventral phallomere oval, moderately wide, sclerotised by sclerite L4A. Lobe bl small, oval. Strip of L4A sclerotising bl even smaller than bl as a whole, but very distinct, curved ventro-dorsad across right edge of the phallomere and narrows towards the apex. Only one process sdp present, its base wide and distal half curved almost at right angle, being directed to the right and slightly posteriad in dorsal perspective and also slightly dorsad in lateral perspective. Posterior edge of sdp convex on the left, then concave, then convex again on the right. Distal half of sdp approximately the same length as sdp base’s width, but three times narrower than long. This distal part strongly sclerotised, slightly flattened in antero-posterior direction and its surface sharply divided in the same direction into posterior smooth area and anterior densely spinulated area including rounded apex. Field of spinules reaches the turning point and continues anteriad as simply strongly sclerotised right edge of sdp. Dorsal sclerotisation of sdp by L4A not covering whole sdp, but with medial membranous evagination almost up to turning point. However, along the right edge it extends even beyond base of sdp, and along left edge of ventral phallomere it reaches process pda as narrow band. Pda expressed only as a lobe, with surface between it and articulation A1 gently concave. A1 simple. Apophysis swe moderately wide and very distinct. Sclerite L4B convex, undulated, and relatively narrow.

Process paa simple, moderately long, directed to the left, but gently curving anteriorly. Edge pba with only one process, presumed to be afa. Afa membranous, moderately sized, bulbous. Pouch pne narrow and gently S-shaped in its anterior part, its posterior and ventral walls sclerotised by sclerite L1. L1 roughly triangular, widened in its right part and sclerotising area of pba immediately anteriad of afa as well as area to the left of afa (on pne plane) but not afa itself. Articulation A2 very wide, articulation A4 absent. Sclerite L2 elongated, with narrow left arm, approximately square right arm and slightly twists along posterior wall of paa leaving dorsal surface of paa weakly sclerotised.

Right phallomere triangular, with strongly concave left edge. Lobe fda covered by short, not very sparse setae within depressions at apex, and sclerotised by sclerite R1A dorsally and along the edges. Arm bm simple, flat. Gap between sclerites R1A and R1B apparent, narrow. Apophysis pia long, partially sclerotised by R1A and in the sclerotised part with slightly uneven edge on macroscale, tuberculate on microscale. Apophysis pva claw-shaped, sclerotised by sclerite R1D. Groove lge very long and narrow, sclerotised by R1B. Sclerite R3 relatively short, axe-shaped, groove age very wide.

**Female.** Unknown.

***Measurements (mm).***TL = 42.7, HW = 4.3, PL = 9.2, PW = 3.0, PnW = 1.4, PzL = 3.1, MzL = 6.1, TgL = 29.3, CfW = 1.1, AL = 26.9, PCL = 6.3, PFL = 8.5, PTL = 4.6, PtL = 5.5, MsFL = 9.1, MsTL = 7.8, MstL = 6.3, MtFL = 10.5, MtTL = 10.0, MttL = 8.5

***Colouration.*** Body pale greenish to brownish with irregular, brownish patches scattered across its surface. Pronotum lighter and more monochrome, with two barely contrasting lateral bands anterior of supracoxal sulcus. Posterior surfaces of prothoracic coxa, femur, and tibia each with two or three darkened bands with highly irregular edges. Meso- and metathoracic legs also with two or three indistinct darkened bands, but only on femur and tibia. Forewing beige with large and small, irregular, brown patches across its surface and interrupted darkened areas along the main veins. Hindwing subhyaline, with darker patches present on apical lobe. Abdomen with longitudinal median stripe paler than lateral ones.

## Discussion

*Arria muscoamicta* sp. nov. lives in evergreen mossy forests at high elevations of approximately 1,200 m above sea level in Thailand. The dominant trees of the region include oaks and chestnuts such as *Lithocarpus*, *Quercus*, and *Castanopsis*, which are covered by bryophytes and epiphytes (Smitinand 1968). The climate of the region (data recorded at the Khao Kheow Weather Observing Station) includes relatively low and consistent air temperatures throughout the year. For example, the annual average temperature between 2017–2020 was 20.35 °C, December (average between 2017–2020) was the coldest month (18.15 °C), while the hottest month was May (average between 2017–2020) with a temperature of 21.77 °C. The relative air humidity is high but fluctuates between 67% and 99%; the lower humidity levels are observed during November and March, which was when the holotype and the male nymph were collected, respectively. It would seem that nymphs of *A. muscoamicta* sp. nov. require a relatively low temperature for their development, as all of our nymphs died after being relocated to a laboratory room with ambient temperature of approximately 30 °C and without air conditioning. The male nymph was collected by searching mossy trees in the vicinity of the holotype collection point, and it is at this stage especially that the mossy-camouflaging morphological peculiarities are apparent (Fig. 1C). In addition to even more patchy colouration than in adult male, all abdominal tergites possess posteromedial lobe similar to those in nymphs and females of *A. leigongshanensis*.

Schwarz and Roy (2018) synonymised *Palaeothespis* Tinkham, 1937 and *Pseudothespis*Mukherjee et al., 1995 with *Arria*, noting that, with respect to the number of foreleg spines, shape, and tuberculation of the head and pronotum, and the shape of male tegmina and of the abdominal lobes in females, *Arria* and *Pseudothespis* fall into the range described for *Palaeothespis*. While this statement is true, there are significant morphological differences between different species of *Arria* (sensu Schwarz and Roy 2018), involving presence/absence of the pronotal tubercles, shape of the pronotum, shape of the apical lobe of the hind wing, and number of various foreleg spines. In addition, the species composition of and distinction between *Arria* and the closely allied genus *Sinomiopteryx* Tinkham, 1937, are currently somewhat ambiguous. For example, *A. sticta* and *A. pallida* are significantly more similar in morphology to *S. brevifrons* and *S. yunnanensis* Xu, 2007 than to the other *Arria* species, in being united by the gently oval edges of the supracoxal dilatation, narrow tegmina, and pointed, lancet-shaped apical lobe of the hindwing. A specimen from Thailand investigated by Schwarz and Roy (2019), whose genitalia are depicted in their work as “*Sinomiopteryx* sp.”, also belongs to the latter group. Another specimen from that group from Laos (in the collection of SMNK) with almost identical genitalia was examined by the second author. The genitalia of these specimens are strikingly different from those of *A. muscoamicta* sp. nov. At the same time, the abovementioned species do not share with *S. grahami* Tinkham, 1937, the type species of *Sinomiopteryx*, some of its most prominent characters, such as very wide forewings with strongly curved main veins and large space between R and ScP, truncated apex of hindwings and somewhat more defined supracoxal dilatation.

While this work was in peer review, another paper has been published (Wang et al. 2021) which has clarified some of the abovementioned issues, specifically by synonymizing *S. yunnanensis* with *A. pallida* and by transferring *S. brevifrons* to *Arria*. Wang et al. (2021) also suggested that species with the terminal lobe near the distal process of the ventral phallomere (as in the abovementioned group that includes *A. sticta*, *A. pallida*, etc.) should belong to *Arria*, while those without it (including *A. muscoamicta* sp. nov.) should be assigned to *Sinomiopteryx*. Unfortunately, the authors were not able to study the type species of both genera to justify this diagnostic character, and the other characters listed by them are inconsistent within the suggested groupings, leaving the problem still unresolved.

*Arria muscoamicta* sp. nov. is similar to the type species of *Arria*, *A. cinctipes*, in so many respects (e.g., the shape of pronotum and the presence of metazonal tubercles) that we consider our combination to remain a valid one. However, as shown above, the taxonomy of the tribe Arriini as a whole needs revision. This would require genital preparation of all holotypes, currently deposited in the museums of USA (1 species), Sweden (1 species), India (1 species), and China (the remainder). Molecular and ecological data might also provide important insights. The discovery of additional new species in this enigmatic and poorly known group of mantodeans is also highly likely.

## Supplementary Material

XML Treatment for Arria muscoamicta
